# Enzymatic quorum quenching increases antibiotic susceptibility of multidrug resistant *Pseudomonas aeruginosa*

**Published:** 2011-03

**Authors:** S Kiran, P Sharma, K Harjai, N Capalash

**Affiliations:** 1 Departments of Biotechnology; 2 Mirobiology, Panjab University, Chandigarh

**Keywords:** Multidrug resistance, *Pseudomonas aeruginosa*, AHL Lactonase, Biofilm, Antibiotic Susceptibility, antimicrobial therapeutics

## Abstract

**Background and Objectives:**

There is increasing emergence of multidrug resistant *Pseudomonas aeruginosa* (MDR*PA*) strains and drug resistance is positively-correlated with biofilm-forming ability. Since about 10% of *P. aeruginosa* genome is controlled by quorum sensing (QS), alteration in its antibiotic susceptibility by targeting QS was the focus of the present study.

**Materials and Methods:**

One day biofilms of PAO1 and three urinary tract infection MDR*PA* isolates (PA2, PA8 and PA18) were formed in 96-well microtiter plate. Biofilms were exposed to concentration gradient of ciprofloxacin and gentamicin to obtain Minimum Biofilm Eradication Concentration (MBEC) by direct enumeration method. Susceptibility of 24 h biofilms was evaluated by treatment with ciprofloxacin and gentamicin *per se* and in combination with lactonase. The effect was also examined on 72 h biofilms by Scanning Electron Microscopy.

**Results:**

Lactonase treatment did not have any effect on growth of the selected strains but 73.42, 69.1, 77.34 and 72.5% reduction of biofilm was observed after lactonase (1 unit) treatment, respectively. Antibiotics in combination with lactonase (0.3 units) resulted in an increased susceptibility of the biofilm forms by>3.3, 4, 5 and 1.5 folds of MBEC, for ciprofloxacin and>6.67, 12.5, 6 and>2.5 folds, for gentamicin respectively, which could be due to the disruption of biofilm by lactonase treatment as shown by scanning electron microscopy. Also there was significant reduction (p<0.001) in virulence factor production by the strains.

**Conclusion:**

Lactonase treatment increased antibiotic susceptibility of the biofilms of MDR*PA* isolates underscoring the potential of quorum quenching in antimicrobial therapeutics.

## INTRODUCTION

*Pseudomonas aeruginosa* is a leading opportunistic nosocomial pathogen associated with significant morbidity and mortality due to cystic fibrosis, bacteremia, endocarditis, osteomylitis including cranial nerve palsies, gastrointestinal upsets, burns, and ventilator- associated pneumonia among intubated patients (1–4).

This organism is problematic because of impressive genetically encoded mechanisms of intrinsic resistance and the potential to mutate and gain resistance to current antibiotics (5) which primarily corresponds its biofilm forming ability (1). Most notably, fluoroquinolones influence emergence of multidrug resistant strains (6–8). Aminoglycosides also have been documented ineffective even in combination with other antibiotics due to newly evolving resistant strains (9–12). Therefore, ciprofloxacin (fluoroquinolone) and gentamicin (aminoglycoside) have been selected for investigation in the present study.

With the wide application of *in vivo* artificial devices such as contact lens, synthetic valves and artificial joints, *P. aeruginosa* gain access into the body and form biofilm. The established biofilms provides permeabi-lity barrier for antibiotics (13), making the treatment of *P. aeruginosa* infections intractable. Quorum sensing (QS) is known to play a pivotal role in the virulence of *P. aeruginosa* (5). Its QS system is composed of the LasR/I and the RhlR/I signal systems, which produce two typical autoinducers: N-(3-oxo-dodecanoyl)-L-homoserine lactone (OdDHL) and N-butyryl-L-homoserine lactone (BHL), respectively. DNA chip analysis has indicated that *P. aeruginosa* genome has over 300 genes under the control of QS system, including genes related to important virulence factors and biofilm formation (14) but there are equivocal reports about antibiotic resistance being regulated by QS.

The aim of the current study was to test the hypothesis that targeting the dissemination of QS molecules by enzymatic degradation may affect antibiotic susceptibi-lity of *P. aeruginosa* strains.

## MATERIALS AND METHODS

**Strains, media and plasmids**. All bacterial strains and plasmids used in this work along with growth conditions are listed in Table 1. Eight urinary tract infection (UTI) and ten sepsis isolates of *Pseudomonas aeruginosa* isolated from clinical specimens in hospital diagnostic laboratories were subjected to preliminary confirmatory tests using Difco™ Pseudomonas Isolation Agar followed by Kligler Iron Agar and Kovac's oxidase test. The strains were maintained in Luria Bertani (LB) medium, but 2% peptone was used for virulence assays.

**Table 1:** Characteristics of bacterial strains/plasmids.

**Bacterial strains**	**Relevant characteristics/genotype**	**Growth conditions**	**Reference**
*Agrobacterium tumefaciens* NT1(pDCI41E33) (Indicator strain)	*traG*: *lacZ traR* in pDSK519; Km^r^ autoinducer reporter plasmid	Kanamycin (50 µg/ml) at 30°C, AB medium	(15)
*E. coli* DH5α	*deo*R*, end*A1 *gyr*A96*, hsd*R17*(rK*^−^*mK*^+^*), rec*A1*, rel*A1*, sup*E44*, thi-*1**, Δ(*lac*ZYA*- arg*FV169)*, Ø*80*lac*ZΔM15, *F*−	LB medium, 37°C	(16)
• *P. aeruginosa* PAO1	Wild type strain		
• PA2,PA8,PA11,PA18, G1,G9,G10 and G13	Clinical urinary tract infection isolates	LB medium, 37°C	(17) This study
• 2663, 3633, 3751, 3849, 3878, 3882, 4250,4287, 4299 and 4303.	Clinical isolates from sepsis patients		

**Plasmids used**			

pMAL-t-*aiiA*	Expression vector containing lactonase gene (*aiiA*) from *Bacillus thuringiensis*4A3		(18)

**Selection of clinical isolates of** ***P. aeruginosa**.* For biofilm forming ability, *P. aeruginosa* (10^6^ cfu/ml) was seeded into wells of 96-well microtiter plate containing 100µl sterile LB. Un-inoculated LB was taken as control (CW). The plate was incubated at 37°C for 24 h and growth (G) was determined spectrophotometrically at OD_600nm._ Wells were vigorously washed four times with PBS (pH 7.2), crystal violet (1%) was added and the plate was incubated for 10 min. Again the wells were washed and dried thoroughly. 95% ethanol was added to each well and was kept for 10–15 min. The solution from each well was pipetted to fresh wells, biofilm formation (AB) was measured spectrophotometrically at OD_575nm_ and specific biofilm forming index (SBF) was calculated using the formula *AB-CW/G*. The degree of biofilm production was classified in three categories: weak (SBF≤0.5), moderate (0.5>SBF≤1), and strong (SBF>1) (19).

MDR*PA* isolates were screened for lactone production by AHL plate bioassay using *A. tumefaciens* NT1 indicator strain. Briefly, lactones were extracted from overnight cultures with acidified ethyl acetate using 0.01% glacial acetate (2) and were concentrated 1000 times. AB agar plate was overlaid with AB medium containing 0.5% mannitol, X-gal (40 µg/ml), 10% inoculum of *A. tumefaciens* NT1 (pDCI41E33) reporter strain (OD_600nm_ 1.8), 50 µg/ml kanamycin and 0.75% agar. Wells were punched aseptically and the extracted lactones were loaded. Plates were incubated for 24–48 h and presence of lactone was indicated by blue zones around the wells (15).

Kirby-Bauer disc diffusion method was used following NCCLS guidelines for the selection of MDR*PA* isolates (resistant to≥3 different classes of antibiotics) (20). Different antibiotics used were aminoglycosides [amikacin (30 µg), gentamicin (10 µg), kanamycin (10 µg), streptomycin (10 µg), tobramycin (10 µg)], fluoroquinolone [ciprofloxacin (5 µg)], β-lactams [ampicillin (10 µg)] and polyketide [tetracycline (10 µg)].

**Heterologous expression of** ***aiiA*** **in** ***E. coli*** **DH5α and lactonase preparation**. Lactonase gene (*aiiA*) fused to maltose binding protein (MBP) was cloned in expression vector pMAL-c2x (gift from Dr. Walter Fast, University of Texas, Austin) and *E. coli* DH5α (12.5 kV/cm, 200 ω) transformants were selected on ampicillin (50 µg/ml) LB agar plates (16). Expression and production of lactonase was done by method of Thomas et al. (18).

**Lactonase activity**. Lactonase activity was calculat-ed as substrate (µM) degraded/min/µg of lactonase. Residual lactone extracted after incubation of 3-oxo-C_12_ HSL (Fluka, USA) and lactonase at 37°C for 5, 10 and 15 min was added to 96 well plate containing 100 µl of 1:10 diluted overnight grown culture of *A. tumefaciens* NT1 in AB medium supplemented with 0.5% mannitol, 50µg/ml kanamycin and 40 µg/ml X-gal (15) and incubated for 24 h at 37°C. OD was taken at 490 nm (21) and residual lactone was estimated from the standard curve of 3-oxo-C_12_-HSL (0.01 µM-10 µM).

**Biofilm assay**. A static biofilm assay was perform-ed with selected MDR strains of *P. aeruginosa* in 96 well polystyrene microtiter plates. 24 h biofilm was treated in the absence (control) and presence of lactonase gradient (0–1 Unit). After 6 h of treatment at 37°C, excess broth was removed and used to enumerate planktonic growth on LB agar, and the wells were washed four times with sterile PBS. 150µl of PBS was added to all the wells and the microtiter plate with lid was placed in chilled water bath. Sonicator tip was dipped in water-bath and sonicated for 10 sec at 40% power to loosen the biofilm and bacterial cells in biofilms (cfu/ml) were enumerated on LB agar.

**Effect of lactonase on antibiotic resistance of clinical isolates of** ***P. aeruginosa**.* Minimum biofilm eradication concentration (MBEC) of gentamicin and ciprofloxacin was estimated on 24 h biofilms formed by the selected *P. aeruginosa* strains by giving treatment at 0–5 mg/ml and 0–3 mg/ml, respectively.

Concentration of antibiotic at which no viable cell-counts in biofilms were obtained was taken as MBEC. To study the effect of lactonase on antibiotic susceptibility of the selected *P. aeruginosa* isolates, antibiotic treatment was given in absence (control) and presence of lactonase (0.3 Units) for 6 h at 37°C followed by viable counting of biofilm forms as explained above.

**Microscopic analysis**. Scanning electron microsco-pic analysis of the effect of lactonase and gentamicin and the effect of both in combination on PAO1 biofilm was performed. For biofilm formation, cell-suspension (106 cfu/ml) from overnight grown culture at 37°C, 180 rpm was made in AB medium supplemented with 0.3 mM glucose and was used to form 24 and 72 h biofilm on cover-slips [in four sets: untreated control, lactonase (0.3 units) and antibiotic treatment *per se* and in combination] (22). Then the respective treatment for 8 h at 37°C was given to the established biofilm. Cover-slips were vigorously washed 8–10 times with sterile PBS (pH 7.2, 50 mM) and fixed with 2.5% glutaraldehyde for 3 h. The samples were dehydrated using ethanol gradient, for 15 min each. The samples were mounted onto aluminum stubs, sputter-coated with gold-palladium (1.8 kV and 6 mA for 60 s) and desiccated. The electron micrographs were analyzed by digital scanning electron microscope JSM 6100 (JEOL).

**Effect of lactonase on the production of virulence factors of** ***P. aeruginosa*** ****. Selected strains of *P. aeruginosa* were grown in 2% bactopeptone in presence and absence (control) of lactonase (0.3 Unit) in triplicates. After 16 h incubation at 37°C, 180 rpm; the cell density was recorded at OD_600nm_ and cell free supernatant (CFS) was obtained by centrifugation at 12,000 g for 15 min, 4°C for the estimation of virulence factors.

Pyochelin was estimated in 1.0 ml of CFS by adding 1 ml each of 0.5 N HCl, nitrite molybdate reagent and 1 N NaOH. Final volume was made to 5 ml with distilled water and the absorbance was read at 510 nm (23). Protease activity was checked on 0.1 ml of azocasein (10 mg/ml) dissolved in 50 mM PBS (pH 7.5) (Sigma, USA) at 37°C for 1 h. Yellow colored (azo group) hydrolyzed end-product was spectrophotometrically recorded at 405 nm after centrifugation (12,000 g, 2 min) and results were expressed as OD405 nm/h growth unit (24).

Elastolytic activity was measured on elastin–congo red (ECR). Optical density was taken at 490 nm and elastase activity was expressed as absorbance at 490 nm/mg of ECR/h (25). For pyocyanin estimation, pyocyanin was extracted from CFS with 5:1 v/v of chloroform. The chloroform phase was removed and extracted with 0.2 N HCl. Absorbance was taken at 490 nm. Rhamnolipid production was estimated in 1.0 ml CFS by adding 1N H_2_SO_4_. Absorbance was taken at 490 nm (26).

\Results were statistically analyzed by applying the student's t-test for calculating p- values.

## RESULTS

All clinical isolates were observed to be Gram- negative motile rods. Although all the isolates showed growth on Pseudomonas isolation agar, only PAO1, PA2, PA8, PA11 and PA18 produced pyocyanin after 18 h, however, other isolates produced pyocyanin pigment after 48 h of incubation at 37°C. On KIA medium, no acid production in slant and butt indicated that the isolates fermented neither dextrose nor lactose. Also, all the strains were observed to be oxidase positive; therefore, all the *P. aeruginosa* clinical isolates and PAO1 wild-type strain was subjected to screening for biofilm formation, lactone production and antibiotic resistance.

**Fig. 1:**
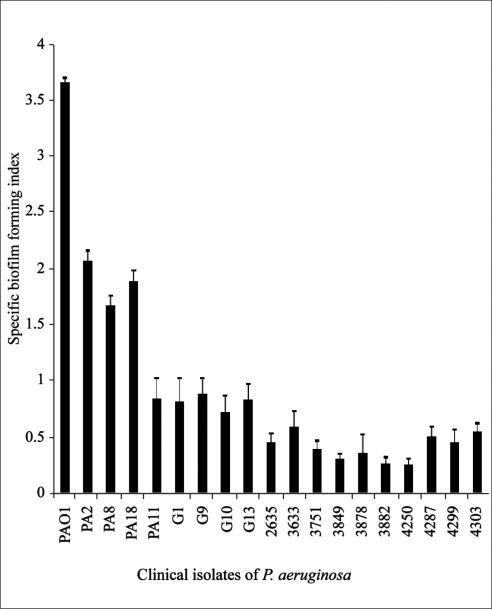
Specific biofilm forming index of *P. aeruginosa* PAO1 strain and clinical isolates±standard deviations.

**Selection of clinical isolates of** ***P. aeruginosa**.* All the clinical isolates were observed to produce lactones, however, the production was higher among UTI isolates as indicated by the intensity of blue zones around the wells (data not shown) but wide variation in biofilm forming abilities was observed (Fig. 1). PAO1 along with PA2, PA8 and PA18 UTI isolates exhibited the strong biofilm forming ability (SBF>1); PA11, G1, G9, G10 and G13 UTI isolates had the moderate (SBF>0.5) and all the sepsis isolates showed the weak biofilm forming ability (SBF≤0.5).

**Fig. 2A:**
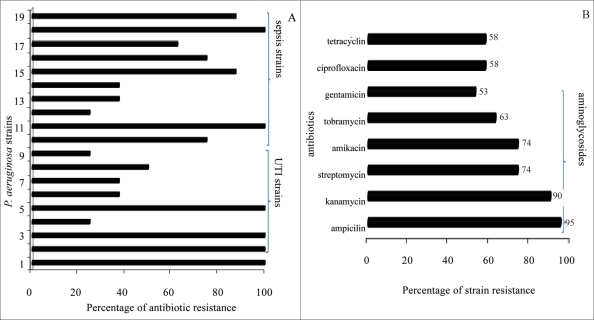
Percent antibiotic resistance by different *P. aeruginosa* strains: 1) PAO1, 2) PA2, 3) PA8, 4) PA11, 5) PA18, 6) G1, 7) G9, 8) G10, 9) G13, 10) 2663, 11) 3633, 12) 3751, 13) 3849, 14) 3878, 15) 3882, 16) 4250, 17) 4287, 18) 4299, 19) 4303.**fig. 2B**. Percent strain resistance to different antibiotics.

The results of antibiotic disc diffusion assay showed multi-drug resistance among three urinary tract infection (UTI) strains (PA2, PA8, and PA18) and six sepsis isolates (2635, 3633, 3751, 3882, 4299 and 4303) (Fig. 2A). PAO1, PA2, PA8, PA18, 3633 and 4299 showed 100% antibiotic resistance against the set of antibiotics used. Beside wide variation in antibiotic resistance levels of the isolates, the whole spectrum of commonly administered antibiotics also showed variable (53–95%) and high percentage (> 50%) of strain resistance (Fig. 2B). Majority of the strains (95%) showed resistance to ampicillin followed by aminoglycosides (71%). Among aminoglycosides, frequency of resistance to antibiotics followed the order: kanamycin (90%)>streptomycin and amikacin (74%)>tobramycin (63%)>gentamicin (53%). 58% strains were resistant to ciprofloxacin and 53% against tetracycline (Fig. 2B).

**Fig. 3:**
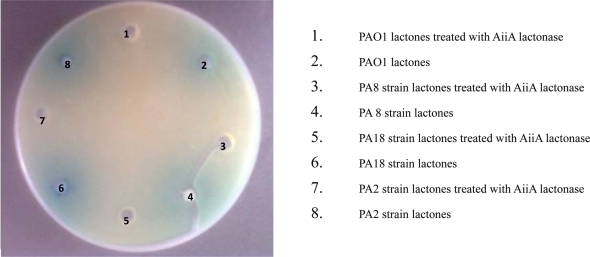
Effect of lactonase on lactones producted by *P. aeruginosa* PAO1 strain and three UTI clinical isolates.

Since PAO1, PA2, PA8 and PA18 strains exhibited strong lactone production; biofilm formation and antibiotic resistance, these were selected for the further investigation. Also, the AHL molecules produced by these selected *P. aeruginosa* strains were observed to be completely degraded by AiiA lactonase (Fig. 3).

**Effect of lactonase on biofilm formation**. Lactonase (1 unit) treatment significantly (p<0.01) reduced biofilm formation by 73.5, 68.8, 76.8 and 70.7% in PAO1, PA2, PA8 and PA18 strains, respectively (Table 2a) and the effect was found to be concentration dependent. There was an increase in the planktonic cell count in the medium surrounding the treated biofilm (Table 2b) which increased with the increase in lactonase concentration (0–1 units). The highest transformation of biofilm forms to planktonic forms (71.2%) due to lactonase (1Unit) treatment was observed for PA2 strain and the least was observed for PAO1 strain of *P. aeruginosa* (63.42%) (Table 2b).

**Table 2a:** Effect of lactonase on biofilm formation of *P. aeruginosa* isolates

Lactonase (Units)	% reduction in log_10_ cfu/ml counts of biofilm after lactonase treatment±SD			
	
	PAO1	PA2	PA8	PA18
0	0±0.065	0±0.97	0±0.47	0±0.17
0.05	2.7±1.01	4.16±0.74	4.59±0.92	7.73±1.3
0.1	25.7±0.17	9.36±0.14	13.8±0.61	24.79±0.8
0.2	38.87±0.27	21.6±0.63	23.55±0.84	34.1±0.62
0.3	49.84±0.09	52.27±0.62	46.6±0.16	47±0.51
0.4	51.25±0.18	53.62±0.81	48.9±0.71	49.3±0.93
0.5	56.52±0.65	56.38±1.09	51.08±0.64	52.99±0.65
0.6	58.24±0.09	57.51±0.72	53.34±0.53	54.67±0.42
0.7	61.53±0.29	58.266±0.65	55.11±0.19	57.96±0.84
0.8	65.12±0.83	63.03±0.82	66.95±0.71	61.97±0.91
0.9	68.14±1.56	69.2±0.37	71.13±0.19	70.32±0.21
1.0	73.42±0.79	69.1±0.61	77.34±0.92	72.5±0.19

**Table 2b:** Effect of lactonase on a) biofilm formation; b) planktonic forms of *P. aeruginosa* PAO1 strain and three UTI clinical isolates.

Units of lactonase	% increase in log_10_ cfu/ml counts of planktonic cells after lactonase treatment±SD			
	
	PAO1	PA2	PA8	PA18
0	0±0.065	0±0.065	0±0.065	0±0.065
0.2	8.87±0.17	10.1±0.29	5.8±1.27	9.87±0.79
0.4	20.25±1.18	25.2±0.28	19.26±0.18	25.25±0.48
0.6	38.24±1.09	42.14±0.39	28.27±0.09	36.24±0.49
0.8	45.12±0.73	55.16±0.3	35.32±0.83	42.12±1.13
1	63.42±0.29	71.2±0.7	69.41±0.79	65.42±0.13

**Figs. 4a-d:**
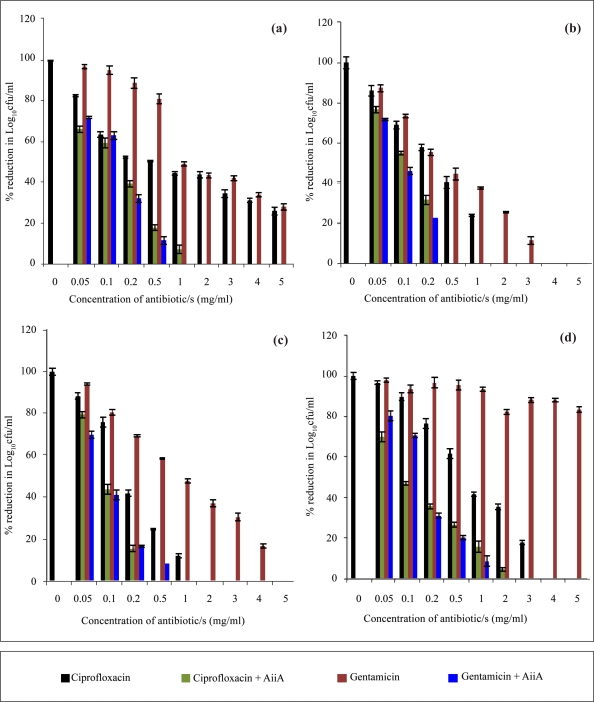
Effect of lactonase treatment on antibiotic resistance of *P. aeruginosa* strains a) PAO1 wild-type b) PA2 c) PA8 d) PA18.

**Effect of lactonase on Minimum Bactericidal Eradication Concentration**. 24 h biofilms of selected MDR strains of *P. aeruginosa* showed different levels of reduction in MBECs (Fig. 3a-3d) against ciprofloxacin and gentamicin when treated in the presence of 0.3 units of lactonase. MBECs of both ciprofloxacin and gentamicin could not be achieved even at 5000 µg/ml, in case of PAO1 strain but with lactonase treatment, MBEC for both the antibiotics was achieved at 1500 µg/ml and 750 µg/ml, respectively (Fig. 4a). Lactonase treatment thus potentiates the antibiotic efficiency because biofilm eradication could be achieved at sub-MBEC levels for both gentamicin and ciprofloxacin.

MBEC of ciprofloxacin and gentamicin against MDR *PA2* isolate of *P. aeruginosa* was 2000 and 4000 µg/ml, respectively, which reduced to 500 µg/ml and 300 µg/ml in presence of lactonase resulting in 4 and 12.5 folds reduction in MBECs, respectively (Fig. 4b).

Gentamicin showed higher MBEC against all the four strains of *P. aeruginosa*. For PA8 strain, MBEC for ciprofloxacin and gentamicin was 2000 µg/ml and 5000 µg/ml, which is approximately 100 times the permissible dosage prescribed for human subjects. Lactonase treatment reduced the MBEC values by 5 and 6 folds, respectively (Fig. 4c). PA18 strain also showed high resistance against both ciprofloxacin (4000 µg/ml) and gentamicin (>5000 µg/ml), however, lactonase treatment resulted in reduction of MBEC levels by 1.5 and>2.5 folds, respectively (Fig. 4d).

**Fig. 5 (a-f):**
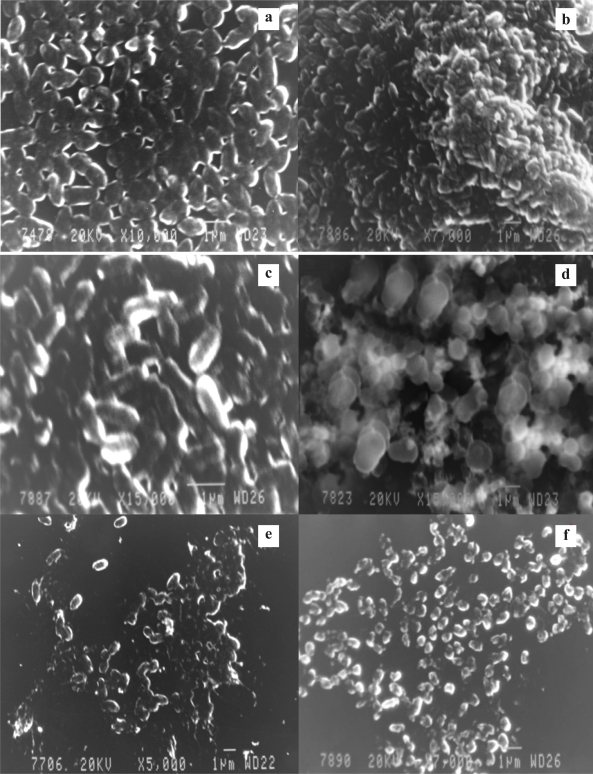
SEM of biofilm formed by PA2 isolate of *P. aeruginosa* on glass surface using Scanning Electron Microscope (Model JSM6100-JEOL). Fig. a) 24 h biofilm; b, c) 72 h biofilm; d) gentamicin (100 µg/ml) treated 72 h biofilm; e) lactonase (0.3 units) treated 72 h biofilm; f) gentamicin (100 µg/ml) and lactonase (0.3 units) treated 72 h biofilm

Microscopic examination of electron micrographs showed multiple cells in contact with one another and irreversibly attached to substratum, with no pili i.e. motility was ceased in attached cells (Fig. 5a) in 24 h biofilm of PA2 isolate. At some fields, cell-clusters had initiated progressive layering. Mushroom and pillar-like structures were visualized in 72 h biofilm (Fig. 5b, 5c) which persisted in gentamicin (sub-MBEC) treated 72 h biofilm but it resulted in altered cell morphology i.e. the cells turned spherical (Fig. 5d). Clustering and mushroom-like structures were absent when treatment was given with lactonase *per se* and in combination with gentamicin, respectively (Fig. 5e, 5f).

**Fig. 6:**
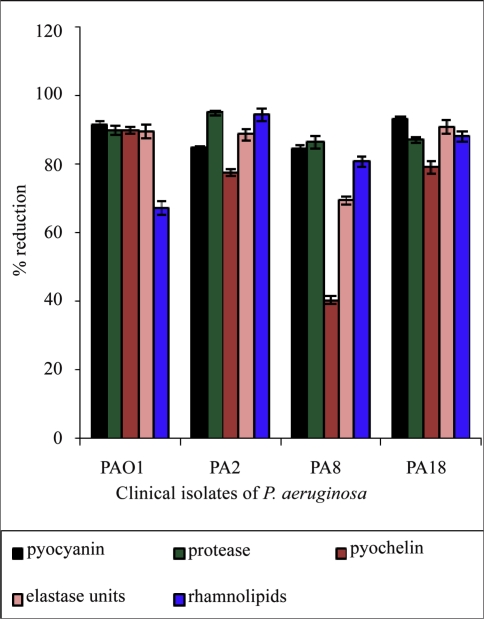
Effect of lactonase on virulence factors of *P. aeruginosa* PAO1 strain and three selected UTI clinical isolates (PA2, PA8 and PA18.)

Also, there was an significant decrease (p<0.001) in the virulence factors after lactonase (0.3 U) treatment. Pyocyanin was reduced in range of 85–93%, protease activity by 86–95%, elastase activity by 69–91% and pyochelin secretion by 40–90% along with rhamnolipids production in the range of 67–94% in different MDR strains of *P. aeruginosa* (Fig. 6).

## DISCUSSION

Intriguing successful reports of QS inhibitors like quorum quenching (QQ) enzymes and synthetic chemicals in controlling virulence have underscored the validity of QQ strategy against numerous human and plant pathogens. There is very little data on its application in combating drug resistance. With the widespread use of extended-spectrum antibiotics, clinical evolution of MDR*PA* strains is rising (27–30) which contributes to the persistence of biofilm infections that necessitates early multidisciplinary control interventions.

To analyze the effect of lactonase on antibiotic susceptibility of clinical isolates of *P. aeruginosa*, the sampling was focused on UTI and sepsis isolates because high rates of mortality (70%) are due to UTI, burn wounds, sepsis and resultant endocarditis (3, 4). The clinical isolates were assayed for lactone production, biofilm formation and multidrug resistan-ce prior to selection for further analytical studies because clinical isolates could be QS mutants which do not produce AHL auto-inducers and have reduced expression of virulence traits (31). During selection, it was observed that UTI isolates were strong biofilm and lactone producers *vis-à-vis* sepsis isolates. This striking feature couldn't be explained because both adherent and invasive forms i.e. UTI and sepsis forms, respectively, are strong biofilm formers (19). Also there is no comparative analysis yet reported for the variable physiological responses by isolates from different body sites. But the higher lactone production and SBF index of UTI isolates could provide no advantage in multidrug resistance as only three UTI (PA2, PA8 and PA18) and six sepsis isolates (2635, 3633, 3751, 3882, 4299 and 4303) were MDRs along with PAO1 strain. Since, PAO1, PA2, PA8 and PA18 fulfilled all the selection parameters of antibiotic resistance, biofilm and lactone production, these were selected for further investigations.

In an experiment on lactonase effect on biofilms, lactonase was observed to eradicate 24 h biofilms in concentration dependent manner and was independent of the biofilm species. Since biofilms are 100–1,000 times recalcitrant to antibiotics than their planktonic counterparts (2) and reach an accumulation plateau by 24 h (32–34), antibiotic susceptibility in this work was investigated on 24 h biofilms. Although all the biofilms exhibited higher but variable resistance to gentamicin *vis-à-vis* ciprofloxacin but lactonase treated biofilms exhibited enhanced susceptibility to both the antibiotics irrespective of the biofilm species. The higher resistance to gentamicin could be because of delayed transportation of gentamicin across the *P. aeruginosa* biofilm due to more and tighter binding sites (35, 36) which reduces gentamicin activity due to enzymatic degradation during the course of penetration. Also, *P. aeruginosa* biofilms have been reported to display higher resistance to gentamicin *vis-à-vis E. coli* and *Klebsiella* biofilms (36). Although the role of quorum sensing in antimicrobial resistance is not yet clear, this work resolves the role of quorum sensing in antibiotic resistance of *P. aeruginosa* and is confluent with the few of previous studies showing antibiotic resistance due to QS regulated SdiA protein in *E. coli* (37), QS-multi-drug efflux symports in *P. aeruginosa* (38), and AI-2 QS system in *Streptococcus anginosus* (39) but contrary to that of Butler et al. (40) who stated that resistance is directly correlated with density of cells. For further analysis, microscopical examination was performed.

Electron micrographs showed ample microcolonies and typical mushroom and pillar-like structures in 24 h and 72 h biofilm, respectively. The microscopic examination of untreated and gentamicin treated 72 h biofilm showed similar density of bacterial cells but gentamicin treatment caused marked morphological alterations accompanied by rounding of cells. Such morphological alterations of bacterial cells could be due to impairment in synthesis of certain enzymes involved in cell-wall formation as aminoglycosides don't influence the cell wall composition directly (41, 42). Also 72 h biofilm treated with lactonase *per se* and in combination with gentamicin showed abundant scattered cells with rounded cells in the latter. It showed that lactonase enzyme had the significant role in biofilm eradication *vis-à-vis* gentamicin because number of scattered cells was similar in both the samples. These results underscores the potential of lactonase in eradicating the established mature biofilms as compared to previous works that showed efficiency of QS inhibitors, lactonase and acylase enzymes in inhibiting biofilm formation when were allowed to act during inception (32, 43, 44). Thus, manipulating QS pathway of bacterial pathogens may provide an important approach in control of biofilm-associated virulence and development of new anti-bacterial therapeutics.

Furthermore, lactonase treatment significantly reduced (p<0.001) the production of pyocyanin, pyochelin, protease and elastolytic activity which are controlled by *las* QS system and rhamnolipids production by *rhl* QS system (17). Thus, AiiA lactonase quenches all the major lactones (C4-HSL by *rhl* system alongwith 3-oxo-C_12_-HSLs by *las*) produced by *P. aeruginosa* strains that regulate virulence factor expression.

The present study shows promising potential of enzymatic quorum quenching to combat not only the virulence but also uprooting the multidrug resistance
